# Multiple Introductions of Avian Influenza Viruses (H5N1), Laos, 2009–2010

**DOI:** 10.3201/eid1807.111642

**Published:** 2012-07

**Authors:** Stephanie Sonnberg, Phouvong Phommachanh, Tri Satya Putri Naipospos, Joanna McKenzie, Chintana Chanthavisouk, Som Pathammavong, Daniel Darnell, Phetlamphone Meeduangchanh, Adam M. Rubrum, Mahanakhone Souriya, Bounkhouang Khambounheuang, Richard J. Webby, Bounlom Douangngeun, Robert G. Webster

**Affiliations:** St. Jude Children’s Research Hospital, Memphis, Tennessee, USA (S. Sonnberg, D. Darnell, A.M. Rubrum, R.J. Webby, R.G. Webster);; National Animal Health Centre, Vientiane, Laos (P. Phommachanh, P. Meeduangchanh, B. Douangngeun);; Food and Agriculture Organization of the United Nations, Rome, Italy (T.S.P. Naipospos, J. McKenzie, C. Chanthavisouk, S. Pathammavong); and; Ministry of Agriculture and Forestry, Vientiane (M. Souriya, B. Khambounheuang)

**Keywords:** H5N1, influenza, Laos, surveillance, viruses, domestic poultry, avian influenza virus

## Abstract

Avian influenza viruses (H5N1) of clades 2.3.4.1, 2.3.4.2, and 2.3.2.1 were introduced into Laos in 2009–2010. To investigate these viruses, we conducted active surveillance of poultry during March 2010. We detected viruses throughout Laos, including several interclade reassortants and 2 subgroups of clade 2.3.4, one of which caused an outbreak in May 2010.

Since 2003, highly pathogenic avian influenza virus (H5N1) has spread from southern China throughout Southeast Asia and to Europe and Africa (*1**,**2*). Since 2003, Laos has experienced outbreaks of clade 1 (2003), clade 2.3.4 (2006, 2007, 2008, 2009, 2010), and clade 2.3.2 viruses (twice in 2008) (*3**,**4*). Active surveillance of domestic ducks and chickens in Laos has been limited, but serum antibodies against subtypes H5 and H9 have been detected in ducks. In addition, subtype H5N1 virus was isolated from healthy ducks in 2006, and subtype H3N8 virus was detected in 2007 (*4**,**5*). To explore the diversity, extent, and endemicity of avian influenza viruses in Laos, we conducted a survey of healthy domestic poultry throughout the country in March 2010.

## The Study

Serum samples were collected in 9 of 17 provinces in Laos from healthy ducks and chickens in live-bird markets, village backyard flocks, and layer duck farms. Cloacal, tracheal, and environmental (fecal and water) swab specimens were also collected and placed immediately in transport medium (Figure). Swab specimens were screened in pools of 4 by using a real-time reverse transcription PCR for the matrix (M) gene segment (*6*). Positive pools were reextracted individually, retested, tested for hemagglutinin 5 (H5) by real-time reverse transcription PCR (*7*), and injected into 10–11-day-old embryonated chicken eggs.

**Figure:**
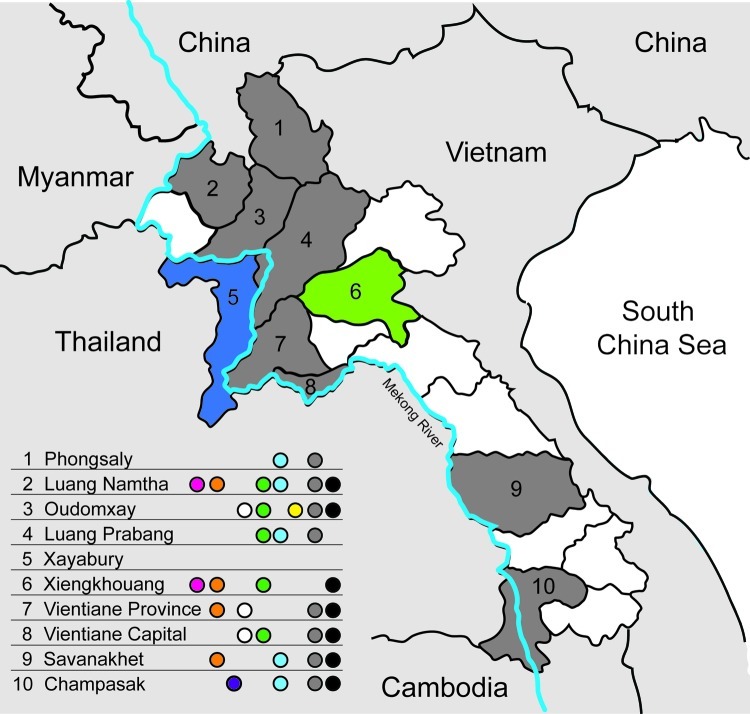
Areas sampled and location of subtyped avian influenza viruses (H5N1), Laos, 2009–2010. Provinces that had previous outbreaks of highly pathogenic avian influenza and were part of the survey are indicated in gray, the province that had a previous outbreak but was not part of the survey is indicated in blue, and the province that had not had an outbreak but was part of the survey is indicated in green. Colored dots indicate presence of viruses: light blue, anti-H5 (clade 2.3.4); gray, anti-H9 lineage G1; red, clade 2.3.4.1; orange, clade 2.3.4.2; green, anti-H5 (clade 2.3.2); black, anti-H9 lineage Y280; white, anti-H4; yellow, anti-H6; purple, clade 2.3.2.1 or virus-specific antibodies.

Sequencing was conducted by using an Illumina (San Diego, CA, USA) platform (swabs and isolates) (*8**,**9*) and conventional Sanger sequencing (isolates). Illumina reads were first mapped to a database of publicly available human, avian, and swine influenza virus reference sequences from the Western Hemisphere Americas and Eurasia, and then remapped against references with the highest number of reads and best average coverage. Final average coverage varied between samples and segments with ranges of 123–24,163 (9 samples), 21–18,671 (12 samples) and 9–436 for sample A/duck/Lao/670/10. Isolate genotypes were verified by using Sanger sequencing. Mixed infections could not be excluded for direct sequencing in the absence of an isolate.

Phylogenetic analysis (ClustalW [http://www.clustal.org/], neighbor-joining analysis, 1,000 bootstrap tests, maximum composite likelihood, pairwise deletions) was conducted by using MEGA version 5.02 (*10*). Sequences are available in GenBank (CY098294–CY098334, CY098336–CY098340, CY098342–CY098368, and CY098370–CY098464). Serum samples were screened by using an ELISA (FlockChek MultiS Screen; IDEXX Laboratories, Westbrook, ME, USA), and antibody-positive serum samples were tested by using a hemagglutinin inhibition assay for subtypes H3, H4, H5 (clades 2.3.2 and 2.3.4), H6, and H9 (lineages G1 and Y280) as described (*11*).

**Figure A1:**
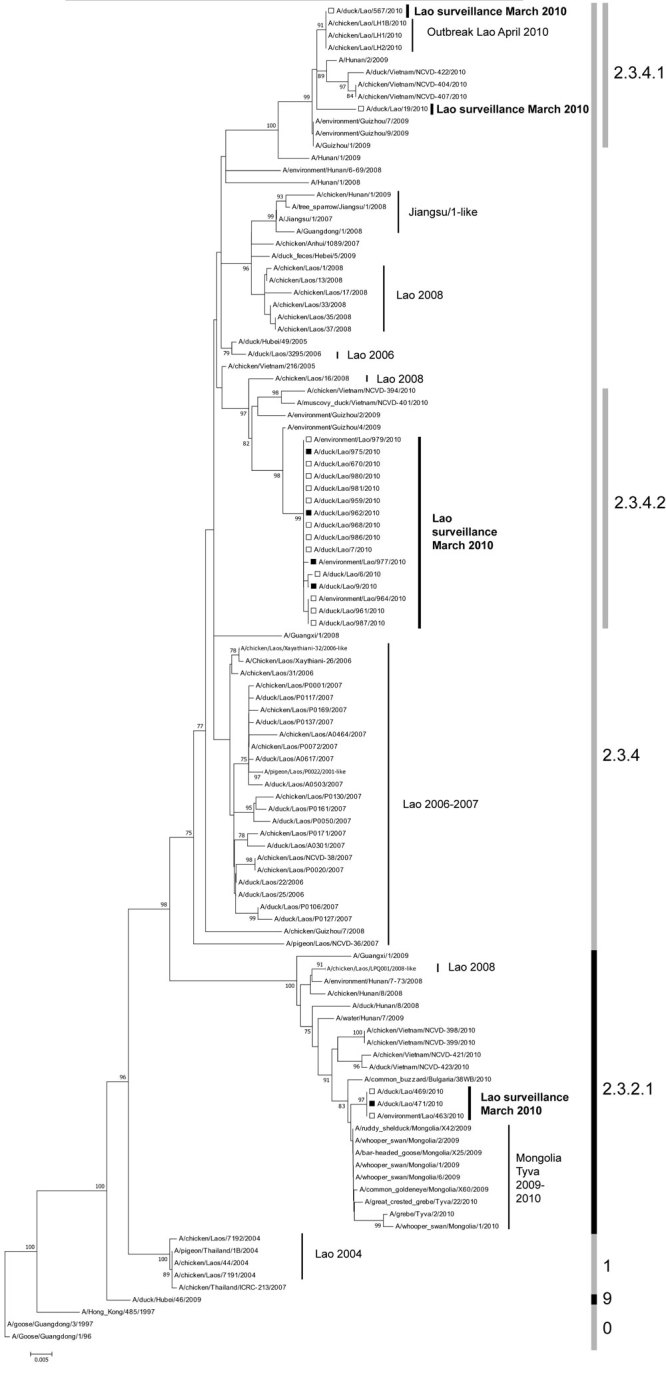
Phylogenetic relationships of hemagglutinin 5 (H5) gene segments of avian influenza viruses (H5N1), Laos, 2009–2010. The tree was rooted in A/goose/Guangdong/1/1996 (nt 91–919, H5 numbering). Surveillance sequences are indicated in **boldface** and marked with black (isolates) or open (direct sequences) squares. Bootstrap values >75 are shown. Clade numbers are shown on the right. Scale bar indicates nucleotide substitutions per site. A/chicken/Laos/Xayathiani-32/2006-like, A/pigeon/Laos/P0022/2007-like, and A/chicken/Laos/LPQ001/2008-like viruses each represent 3 sequences with identical hemagglutinins. Reference viruses were obtained through EpiFlu of the Global Initiative on Sharing All Influenza Data and provided by the following laboratories in addition to sequences already in the public domain: A/chicken/Vietnam/NCVD-407/2010, A/chicken/Vietnam/NCVD-394/2010, A/chicken/Vietnam/NCVD-398/2010, A/chicken/Vietnam/NCVD-399/2010, A/duck/Vietnam/NCVD-422/2010, A/chicken/NCVD-404/2010, A/Muscovy_duck/Vietnam/NCVD-401/2010, A/chicken/Vietnam/NCVD-421/2010 A/duck/Vietnam/NCVD-423/2010 (original laboratory: National Centre of Veterinary Diagnostics, Hanoi, Vietnam; submitting laboratory: Centers for Disease Control and Prevention, Atlanta, GA, USA); A/Hunan/2/2009, A/Hunan/1/2009, A/Guizhou/1/2009, A/Guangxi/1/2009, A/environment/Guizhou/2/2009, A/environment/Guizhou/4/2009, A/environment/Guizhou/7/2009, A/environment/Guizhou/9/2009, A/water/Hunan/7/2009A/Guangxi/1/2008, A/Guangdong/1/2008, A/Hunan/1/2008, A/duck feces/Hebei/5/2009 (original and submitting laboratory: World Health Organization Chinese National Influenza Center, Beijing, China), Jiangsu/1-like–A/tree_sparrow/Jiangsu/1/2008–like viruses.

During March 2010, a total of 3,695 swab specimens were collected (1,928 duck and 279 chicken cloacal samples, 446 duck tracheal samples, 675 fecal samples, and 367 water samples). M gene prevalence was 4.0% (ducks), 1.8% (chickens), and 0.3% (environment samples). Five isolates were obtained (Figure A1). All M gene–positive swab specimens were collected in 13 locations (8 backyards, 2 markets, and 3 farms) (Table 1; Figure) Sample protection was suboptimal, and only 21 samples could be subtyped by real-time RT-PCR and sequencing (Table 1).

**Table 1:** Genotypes of 21 surveillance samples of avian influenza virus (H5N1) and outbreak virus A/chicken/Lao/LH1/2010, Laos, 2009–2010*

Sample ID	Sample type	Location†	Sampling site	Gene‡							
				PB2	PB1	PA	H5§	NP	N1	M	NS
**A/ck/LH1**	Chicken	Vientiane	Farm				2.3.4				
210317	Duck	Xiengkhouang-1	Market		NA	NA	2.3.4				
210265	Duck	Luang Namtha-1	Backyard		NA	NA	2.3.4				NA
210281	Env	Champasak-1	Market		NA	NA	2.3.2				NA
210287	Duck	Champasak-1	Market		NA		2.3.2				
**210289**	Duck	Champasak-1	Market				2.3.2				
210252	Duck	Luang Namtha-1	Backyard				2.3.4				
210253	Duck	Luang Namtha-1	Backyard				2.3.4				
**210255**	Duck	Luang Namtha-1	Backyard				2.3.4				
210358	Duck	Xiengkhouang-2	Backyard				2.3.4				
210360	Duck	Xiengkhouang-2	Backyard			NA	2.3.4				
**210361**	Duck	Xiengkhouang-2	Backyard				2.3.4				
210363	Env	Xiengkhouang-2	Backyard				2.3.4				
210367	Duck	Xiengkhouang-2	Backyard			NA	2.3.4				
**210374**	Duck	Xiengkhouang-2	Backyard				2.3.4				
**210376**	Env	Xiengkhouang-2	Backyard				2.3.4				
210378	Env	Xiengkhouang-2	Backyard				2.3.4				
210379	Duck	Xiengkhouang-2	Backyard				2.3.4				
210380	Duck	Xiengkhouang-2	Backyard				2.3.4				
210385	Duck	Xiengkhouang-2	Backyard				2.3.4				
210386	Duck	Xiengkhouang-2	Backyard				2.3.4				
210349	Duck	Savanakhet-1	Farm				2.3.4			NA	

*Boldface indicates outbreak virus isolate and closely related isolates. ID, identification; PB, polymerase basic; PA, polymerase acidic; H, hemagglutinin; NP, nucleoprotein; N, neuraminidase; M, matrix; NS, nonstructural; NA, not available; env, environmental. †Identical numbers indicate same sampling site. ‡Pink shading, A/Guizhou/1/2009-like clade 2.3.4.1; blue shading, A/whooper swan/Mongolia/6/2009-like clade 2.3.2.1; purple shading, A/tree_sparrow/Jiangsu/1/08-like clade 2.3.4; gray shading, A/environment/Guizhou/4/2009-like clade 2.3.4.2. §Clade is indicated.

Phylogenetic analysis identified 3 groups of viruses: clades 2.3.2.1, 2.3.4.1, and 2.3.4.2 (Figure A1). Two samples were closely related to A/chicken/Lao/LH1/2010–like virus (outbreak in Vientiane in April–May 2010) and to A/chicken/Laos/C100209–194-PTK/2009–like virus (outbreak in Phongsaly in February 2009) (clade 2.3.4.1) (Figure A1; Table 1). These viruses were closely related to A/Guizhou/1/2009 and A/chicken/Vietnam/NCVD-404/2010 (Figure A1).

Sixteen surveillance viruses of clade 2.3.4.2 were highly homogeneous (Figure A1; Table 1) and closely related to A/environment/Guizhou/4/2009 and A/chicken/Vietnam/NCVD-394/2010–like viruses, although they contained the polymerase basic 2 (PB2) gene of a clade 2.3.2.1 donor virus and were therefore interclade reassortants (Figure A1; Table 1). The genotype of the 4 isolates was verified by using Sanger sequencing. The PB2 gene was most closely related to A/grey_heron/Hong_Kong/1046/2008–like viruses. These reassortants were detected in 3 locations in northern (backyard), central (backyard), and southern (farm) Laos, suggesting multiple introductions of reassortants into Laos (Figure A1; Table 1).

Three clade 2.3.2.1 viruses were detected in 2 ducks and 1 environmental sample (same trader). These viruses were interclade reassortants. Two (1 isolate and 1 direct sequence) contained 6 or 7 segments that were A/whooper swan/Mongolia/6/2009 like (clade 2.3.2.1), and the nucleoprotein gene was A/tree_sparrow/Jiangsu/1/08 like (clade 2.3.4). The environmental sample (direct sequence) contained A/whooper swan/Mongolia/6/2009–like hemagglutinin and M genes and A/Guizhou/1/2009–like PB2, nucleoprotein, and neuraminidase genes (clade 2.3.4.1) (Table 1). The genotype of the isolate was verified by using Sanger sequencing. Hemagglutinin segments of the reassortants were identical (100% nt identity). This identity and the source of the 3 samples (1 trader) suggest that reassortment occurred recently, likely in Laos.

Antibody titers to H5 and H9 and a subtype H3N8 virus isolate have been reported in Laos (*4**,**5*). Subtypes H4 and H6 also circulate in this region (*1**,**2**,**12*). For this study, 2,148 serum samples (1,899 from ducks, 200 from chickens, and 49 from unspecified species) were collected and 267 antibody-positive (seroprevalence 14%) duck and 15 (7.5%) chicken serum samples were detected. Hemagglutination inhibition testing for specific antibodies against H3, H4, H5, H6, and H9 detected all antibodies but to H3 (Table 2). Antibodies against H9 were detected at highest titers and most frequently (1.1% of ducks for each H9 lineage), although some cross-reactivity between G1 and Y280 likely occurred (Table 2). These ducks were most widely distributed (18 [19%] of 97 locations in all 9 provinces). Antibodies against H5 clade 2.3.4 were found at the detection limit in few serum samples (0.4% of ducks in 3 northern and 2 southern provinces). Antibodies against H5 clade 2.3.2 were detected in 0.4% of ducks in 4 northern provinces and the capital of Vientiane.

**Table 2:** Serum HI titers in ducks against avian influenza virus (H5N1) H3, H4, H5, H6, and H9 antigens, Laos, 2009–2010*

Serum sample ID	Location†	Sampling site	HI test antigen subtype‡ (clade or lineage)§					
			H4	H5 (2.3.2)	H5 (2.3.4)	H6	H9 (G1)	H9 (Y280)
790	Phongsaly-1	Backyard	<10	<10	<5	<10	10	<10
797	Phongsaly-1	Backyard	<10	<10	5	<10	<10	<10
798	Phongsaly-1	Backyard	<10	<10	<5	<10	10	<10
925	Phongsaly-2	Backyard	<10	<10	5	<10	<10	<10
11A	Luang Namtha-1	Backyard	<10	<10	<5	<10	10	10
11B	Luang Namtha-1	Backyard	<10	40	<5	<10	<10	10
12	Luang Namtha-1	Backyard	<10	160	<5	<10	<10	<10
14	Luang Namtha-1	Backyard	<10	160	40	<10	<10	<10
15	Luang Namtha-1	Backyard	<10	320	<5	<10	<10	<10
1686	Luang Namtha-2	Backyard	<10	<10	<5	<10	<10	640
1688	Luang Namtha-2	Backyard	<10	<10	<5	<10	10	<10
1696	Luang Namtha-2	Backyard	<10	<10	<5	<10	10	<10
1699	Luang Namtha-2	Backyard	<10	<10	<5	<10	<10	320
1736	Luang Namtha-3	Market	<10	<10	<5	<10	10	<10
757	Luang Namtha-3	Market	<10	<10	10	<10	<10	<10
1314	Oudomxay-1	Backyard	80	<10	<5	80	<10	<10
1319	Oudomxay-1	Backyard	40	10	<5	320	<10	<10
1322	Oudomxay-2	Market	<10	<10	<5	<10	<10	40
1324	Oudomxay-2	Market	<10	<10	<5	<10	<10	10
1325	Oudomxay-2	Market	<10	<10	<5	<10	<10	40
1350	Oudomxay-2	Market	<10	<10	<5	<10	<10	160
1362	Oudomxay-3	Market	80	<10	<5	<10	<10	<10
1372	Oudomxay-3	Market	<10	<10	<5	<10	<10	40
1459	Oudomxay-4	Backyard	<10	<10	<5	640	320	320
1470	Oudomxay-5	Farm	<10	<10	<5	<10	<10	10
24	Luang Prabang-1	Backyard	<10	10	<5	<10	<10	<10
36	Luang Prabang-1	Backyard	<10	<10	<5	<10	10	<10
37	Luang Prabang-1	Backyard	<10	<10	<5	<10	10	<10
38	Luang Prabang-1	Backyard	<10	<10	<5	<10	10	<10
951	Luang Prabang-2	Backyard	<10	<10	5	<10	<10	<10
958	Luang Prabang-2	Backyard	<10	<10	5	<10	10	<10
721	Xiengkhouang-3	Backyard	<10	10	<5	<10	<10	<10
1673	Xiengkhouang-4	Farm	<10	<10	<5	<10	<10	10
1378	Vientiane-1	Backyard	640	<10	<5	<10	20	40
1379	Vientiane-1	Backyard	<10	<10	<5	<10	10	<10
1381	Vientiane-1	Backyard	40	<10	<5	<10	20	<10
1382	Vientiane-1	Backyard	<10	<10	<5	<10	10	<10
1386	Vientiane-1	Backyard	40	<10	<5	<10	<10	40
1389	Vientiane-1	Backyard	<10	<10	<5	<10	10	<10
1393	Vientiane-1	Backyard	40	<10	<5	<10	<10	<10
1526	Vientiane-2	Farm	<10	<10	<5	<10	<10	10
463	Vientiane-1	Market	<10	<10	<5	<10	160	320
464	Vientiane-1	Market	<10	<10	<5	<10	>1,280	>1,280
467	Vientiane-1	Market	<10	<10	<5	<10	160	160
468	Vientiane-1	Market	<10	<10	<5	<10	<10	80
1245	Vientiane-2	Backyard	<10	<10	<5	<10	<10	10
1249	Vientiane-2	Backyard	<10	10	<5	<10	<10	<10
1277	Vientiane-2	Backyard	<10	10	<5	<10	<10	<10
1419	Vientiane-3	Market	20	<10	<5	<10	<10	<10
657	Savanakhet-2	Farm	<10	<10	<5	<10	10	10
990	Savanakhet-3	Farm	<10	<10	10	<10	<10	<10
589	Champasak-2	Backyard	<10	<10	<5	<10	40	<10
606	Champasak-3	Backyard	<10	<10	20	<10	10	10

*H, hemagglutinin; ID, identification; HI, hemagglutination inhibition. †Identical numbers indicate same sampling site. ‡All titers for H3 (A/duck/Laos-Xaythany/A0573/2007) were <10. H4, A/duck/Czech/1956; H5 clade 2.3.2, A/common magpie/Hong Kong/5052/2007; H5 clade 2.3.4, A/duck/Laos/3295/2006; H6, A/turkey/Massachusetts/1965; H9 lineage G1, A/Hong Kong/33982/2009; H9 lineage Y280, A/duck/Hong Kong/Y280/1997. §Values represent HI titers reciprocal to the highest dilution of serum that inhibited haemagglutination of 4 hemagglutination units of antigen by using 0.5% chicken erythrocytes.

Among ducks, 34 (1.7%) were either shedding or had antibodies against avian influenza virus (H5N1), and there was >1 virus-positive or antibody-positive duck in each of the 9 provinces sampled. One village had 36% of sampled ducks exposed to this virus; 18% shed 2.3.4 virus and 18% had antibody to 2.3.2 virus (Table 2). One duck in this village had antibodies against 2.3.2 and 2.3.4 clade viruses. Exposure to 2.3.2 and 2.3.4 viruses was evident in ducks from locations in 3 other provinces (1 district each in Champasak and Xiengkhoung, and several districts in Luang Prabang).

## Conclusions

This study showed that 3 groups of avian influenza viruses (H5N1) were likely introduced into Laos in 2009–2010, one of which resulted in 2 outbreaks (2009, 2010). In all 9 provinces where surveillance was conducted, ducks had been exposed to this virus. Evidence of clades 2.3.2 and 2.3.4 virus activity was detected in 4 provinces. Several interclade reassortants were identified, demonstrating the high genetic mobility of these viruses in the region. Since 2004, Laos has had repeated outbreaks of highly pathogenic avian influenza viruses, which have also been detected in China and Vietnam. There is no evidence that a particular virus lineage has established itself in Laos. The frequency of introduction, diversity, and extent of these viruses in Laos suggests considerable movement of viruses into the country from surrounding territories (China and Vietnam, but not Cambodia) and within the country.
